# Genitourinary

**DOI:** 10.4103/0971-3026.38510

**Published:** 2008-02

**Authors:** B Santh Kumar

A 50-year-old man presented with a history of right testicular pain for one week. On examination, there was tenderness in the right hemiscrotal region. An ultrasound (USG) image of the right testis is shown [Figure 1].

## What is the Diagnosis?

**Figure 1:**
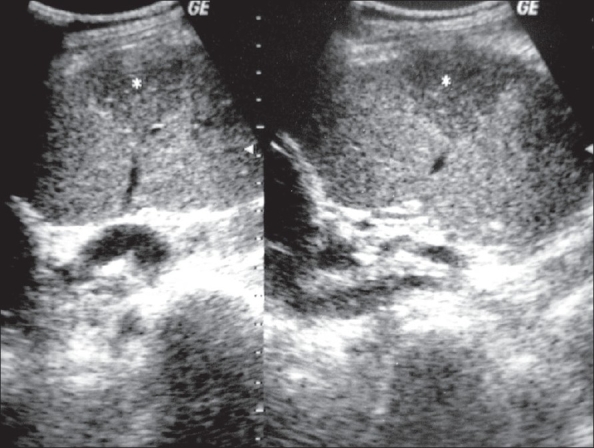
USG of the right testis

**Figure 2:**
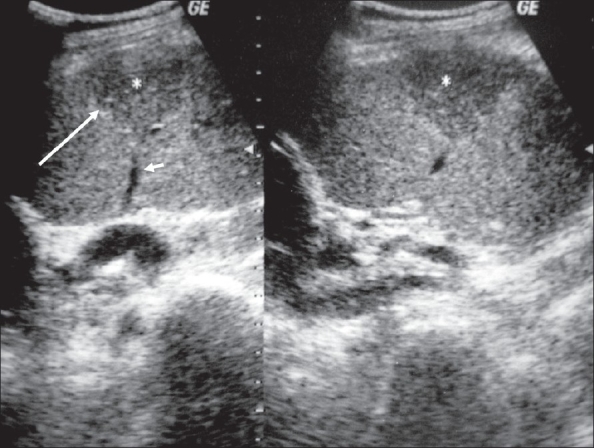
USG of the right testis showing a wedge-shaped lesion (large arrow). A linear hypoechoic vessel is seen terminating at the tip of this wedge-shaped triangular lesion (small arrow)

